# Possible tics diagnosed as stereotypies in patients with severe autism spectrum disorder: a video-based evaluation

**DOI:** 10.1007/s10072-020-04995-1

**Published:** 2020-12-19

**Authors:** Cristiano Termine, Enzo Grossi, Valentina Anelli, Ledina Derhemi, Andrea E. Cavanna

**Affiliations:** 1 Child Neuropsychiatry Unit, Department of Medicine and Surgery, University of Insubria, Varese, Italy; 2 Department of Autism Research, “Villa Santa Maria” Child and Adolescent Neuropsychiatry Rehabilitation Unit, Tavernerio, CO Italy; 3 Michael Trimble Neuropsychiatry Research Group, University of Birmingham & BSMHFT, Birmingham, UK; 4 School of Life & Health Sciences, Aston University, Birmingham, UK; 5 Sobell Department of Motor Neuroscience & Movement Disorders, Institute of Neurology & University College London, London, UK; 6 Department of Neuropsychiatry, National Centre for Mental Health, 25 Vincent Drive, Birmingham, B15 2FG UK

**Keywords:** Autism spectrum disorder, Stereotypies, Tics, Tourette syndrome, Video recording

## Abstract

**Background:**

The association of stereotypies and tics is not rare in children with severe autism spectrum disorder (ASD). The differential diagnosis between stereotypies and tics in this patient population can be difficult; however, it could be clinically relevant because of treatment implications.

**Methods:**

A total of 108 video recordings of repetitive behaviors in young patients with stereotypies in the context of ASD were reviewed by a movement disorders expert and a trainee, in order to assess the prevalence of possible co-morbid tics. The Modified Rush Videotape Rating Scale (MRVS) was used to rate tic frequency and severity.

**Results:**

Out of 27 patients with stereotypies (24 males; mean age 14 years), 18 (67%) reported possible tics. The most frequently observed tics were eye blinking, shoulder shrugging, neck bending, staring, and throat clearing. The mean MRVS score was 5, indicating mild tic severity. The only significant difference between patients with tics and patients without tics was the total number of stereotypies, which was higher in the subgroup of patients without tics (*p* = 0.01).

**Conclusions:**

Expert review of video-recordings of repetitive behaviors in young patients with ASD and stereotypies suggests the possibility of a relatively high rate of co-morbid tics. These findings need to be integrated with a comprehensive clinical assessment focusing on the diagnostic re-evaluation of heterogeneous motor manifestations.

## Introduction

Tics and stereotypies are often reported as repetitive behaviors in patients with autism spectrum disorder (ASD) [1]. Tics are defined as sudden, rapid, recurrent, non-rhythmic movements, and vocalizations [2]. Moreover, tics can be simple (e.g., eye blinking, grunting) or complex (e.g., imitating goal-directed actions, shouting words) [3]. Stereotypies are repetitive, seemingly driven, and apparently purposeless motor behaviors, postures, or utterances. Motor stereotypies are also categorized as simple (e.g., leg shaking) or complex (e.g., hand flapping, body rocking) [4]. Although most stereotypes are mild and perceived as self-soothing; similarly to tics, they can occasionally interfere with daily activities and may result in self-injury.

In terms of clinical phenomenology, stereotypies tend to be more fixed, rhythmic, and prolonged in duration than tics, which are rapid and fluctuating in both intensity and frequency. Stereotypies have a mean age of onset below 3 years, somewhat earlier than tics (5–7 years). Moreover, stereotypies typically occur both in understimulating or overstimulating environments, while active concentration tends to alleviate tics [4]. Finally, unlike stereotypies, most tics are preceded by premonitory urges, subjective sensations of inner tension, which are temporarily relieved by tic expression [5]. It can be notoriously difficult to elicit a reliable history of sensory symptoms associated with hyperkinetic manifestations in younger patients with autistic traits and/or learning difficulties.

The differential diagnosis between tics and stereotypies in children with a diagnosis of ASD who present with heterogeneous repetitive behaviors can pose significant challenges; however, it could be clinically relevant because of treatment implications. For example, pharmacotherapy can be useful in modulating tics, while stereotypies show poor response to medication, although they can benefit from specific behavioral approaches [4, 6]. In the present study, we reviewed video recordings of repetitive behaviors in young patients with ASD and stereotypies in order to evaluate the prevalence of possible tics.

## Methods

The repetitive behaviors of 67 youngsters diagnosed with severe ASD were recorded to build a video database of stereotypies of the patients attending the “Villa Santa Maria” Child and Adolescent Neuropsychiatry Rehabilitation Unit in Tavernerio (Como, Italy). The video clips featured a total of 319 stereotypies: 191 simple plus 128 complexes. A subsample of 108 video recordings with a duration longer than 1 min was subsequently reviewed by a movement disorders expert (CT) and a trainee (VA), in order to assess the possible presence of co-morbid tics. The observed motor and vocal tics were classified as either simple or complex according to their characteristics. The modified Rush Videotape Rating Scale (MRVS) [7] was used to rate the frequency and severity of tics based on careful analysis of the video recordings. Demographic and clinical data, including Autism Diagnostic Observation Schedule, 2nd Edition (ADOS-2) severity scores [8], language function, intellectual disability, and diagnosis of epilepsy, were collected in a database. The collected data were compared to assess possible differences between the groups of patients with co-morbid tics and patients without tics. Statistical analyses included an independent *t* test (for continuous variables), Fisher’s exact test (for categorical variables), and Pearson’s correlation index (for correlation analysis). *P* values below 0.05 were considered significant. The study was approved by the local Ethics Committee (ASST Sette Laghi).

## Results

The demographic and clinical data of the study sample are summarized in Table 1.

**Table 1:** Demographic and clinical characteristics of the study sample

Age (mean, SD)		14 (3)
Male (*n*, %)		24 (89%)
ADOS-2	Calibrated severity index (mean, SD)	8 (2)
ADOS-2 severity index	High (*n*, %)	14 (56%)
	Moderate (*n*, %)	10 (40%)
	Low (*n*, %)	1 (4%)
Language	Absent (*n*, %)	13 (48%)
	Single words (*n*, %)	10 (37%)
	Sentences (*n*, %)	3 (11%)
	Fluent (*n*, %)	1 (4%)
Intellectual disability	Borderline (*n*, %)	1 (4%)
	Mild (*n*, %)	1 (4%)
	Moderate (*n*, %)	8 (30%)
	Severe (*n*, %)	17 (63%)
Epilepsy (*n*, %)		5 (19%)

Out of 27 patients with ASD and stereotypies (24 males; mean age 14 years), 18 (67%) were rated as showing possible tics. Specifically, 6 patients (22%) had multiple motor and simple vocal tics (consistent with a possible diagnosis of Tourette syndrome), 8 (30%) had simple motor tics, 2 (7%) had complex motor tics, and 2 (7%) had both simple and complex motor tics. A total of 85 possible tics were observed, corresponding to 70 different tic types (34 simple motor tics, 23 complex motor tics, 13 simple vocal tics). The most frequently observed tics were eye blinking (8 patients), shoulder shrugging (5 patients), neck bending (4 patients), staring (3 patients), and throat clearing (3 patients). The mean MRVS score was 5 (SD 2), indicating mild tic severity.

Patients with tics and patients without tics had similar demographic and clinical characteristics. The only significant difference was the total number of stereotypies, which was found to be higher in the subgroup of patients without tics (*p* = 0.01). However, according to the correlation analysis (Pearson’s index), there was no significative correlation between the number of stereotypies (or ASD severity, as per ADOS scores) and the number of tics.

## Discussion

Expert review of video-recordings of repetitive behaviors in young patients with ASD and stereotypies suggests the possibility of a relatively high rate of co-morbid tics. Previous studies focusing on the motor phenotypes in ASD populations found prevalence figures of 22% for tics and 8–11% for Tourette syndrome [9, 10]. Our findings show that the prevalence of tics could be considerably higher in the subgroup of young patients with severe ASD and stereotypies. With regard to body areas and types of tics, our results are in line with previous observations indicating that the most common tics involve eyes, face, shoulders, and upper limbs [3]. Moreover, the findings of a recent study in patients with Tourette syndrome showed that 14% of patients from a clinical sample had stereotypies, often in association with ASD, suggesting a shared neurodevelopmental pathway for at least a subgroup of patients [6]. In the present study, the total number of stereotypies was higher in the subgroup of patients without tics, suggesting the possibility that the ASD spectrum might be characterized by multiple atypical neurodevelopmental processes. Our findings highlight the need for a comprehensive assessment of different hyperkinetic manifestations in patients with severe ASD. The segregation of the motor phenotypes would be an important step towards the optimization of treatment interventions, including pharmacotherapy (e.g., administration of alpha-2 agonists for the treatment of milder tics and antidopaminergic agents for more severe tics).

The main limitations of the present study include the relatively small sample size and the highly selected clinical population (video recordings from young patients with severe ASD and motor manifestations recruited at a specialist center). In conclusion, the results of our study suggest that tics might be relatively common in young patients with severe ASD who present with heterogeneous repetitive behaviors. These findings need to be integrated with a comprehensive clinical assessment focusing on diagnostic re-evaluation. In consideration of the possible clinical implications in terms of both diagnostic accuracy and treatment options, further studies on the prevalence and clinical characteristics of both tics and stereotypies are needed to better characterize the motor phenotypes of patients with severe ASD.

## References

[1] Martino D, Hedderly T (2019). Tics and stereotypies: a comparative clinical review. Parkinsonism Relat Disord.

[2] Cavanna AE, Termine C (2012). Tourette syndrome. Adv Exp Med Biol.

[3] Ganos C, Münchau A, Bhatia KP (2014). The semiology of tics, Tourette’s, and their associations. Mov Disord Clin Pract.

[4] Mackenzie K (2018). Stereotypic movement disorders. Semin Pediatr Neurol.

[5] Cavanna AE, Black KJ, Hallett M, Voon V (2017). Neurobiology of the premonitory urge in Tourette’s syndrome: pathophysiology and treatment implications. J Neuropsychiatry Clin Neurosci.

[6] Ubhi M, Achinivu K, Seri S, Cavanna AE (2020). Motor stereotypies in adult patients with Tourette syndrome. Future Neurol.

[7] Goetz CG, Pappert EJ, Louis ED, Raman R, Leurgans S (1999). Advantages of a modified scoring method for the rush video-based tic rating scale. Mov Disord.

[8] Lord C, Rutter M, Luyster RJ, Gotham K, Bishop S (2012). Autism diagnostic observation schedule.

[9] Baron-Cohen S, Mortimore C, Moriarty J, Izaguirre J, Robertson MM (1999). The prevalence of Gilles de la Tourette’s syndrome in children and adolescents with autism. J Child Psychol Psychiatry.

[10] Canitano R, Vivanti G (2007). Tics and Tourette syndrome in autism spectrum disorders. Autism.

